# Effects of online tDCS and hf-tRNS on reading performance in children and adolescents with developmental dyslexia: a study protocol for a cross sectional, within-subject, randomized, double-blind, and sham-controlled trial

**DOI:** 10.3389/fneur.2024.1338430

**Published:** 2024-03-12

**Authors:** Andrea Battisti, Giulia Lazzaro, Cristiana Varuzza, Stefano Vicari, Deny Menghini

**Affiliations:** ^1^Child and Adolescent Neuropsychiatry Unit, Bambino Gesù Children’s Hospital, IRCCS, Rome, Italy; ^2^Department of Human Sciences, LUMSA University, Rome, Italy; ^3^Department of Life Sciences and Public Health, Catholic University of the Sacred Heart, Rome, Italy

**Keywords:** brain stimulation, neurodevelopmental disorders, learning disorders, treatment, tES

## Abstract

**Background:**

Developmental Dyslexia (DD) is a brain-based developmental disorder causing severe reading difficulties. The extensive data on the neurobiology of DD have increased interest in brain-directed approaches, such as transcranial direct current stimulation (tDCS), which have been proposed for DD. While positive outcomes have been observed, results remain heterogeneous. Various methodological approaches have been employed to address this issue. However, no studies have compared the effects of different transcranial electrical stimulation techniques (e.g., tDCS and transcranial random noise stimulation, tRNS), on reading in children and adolescents with DD.

**Methods:**

The present within-subject, double-blind, and sham-controlled trial aims to investigate the effects of tDCS and hf-tRNS on reading in children and adolescents with DD. Participants will undergo three conditions with a one-week interval session: (A) single active tDCS session; (B) single active hf-tRNS session; and (C) single sham session (tDCS/hf-tRNS). Left anodal/right cathodal tDCS and bilateral tRNS will be applied over the temporo-parietal regions for 20 min each. Reading measures will be collected before and during each session. Safety and blinding parameters will be recordered.

**Discussion:**

We hypothesize that tRNS will demonstrate comparable effectiveness to tDCS in improving reading compared to sham conditions. Additionally, we anticipate that hf-tRNS will exhibit a similar safety profile to tDCS. This study will contribute novel insights into the effectiveness of hf-tRNS, expediting the validation of brain-based treatments for DD.

## Introduction

Reading acquisition is a critical milestone in human development – particularly in modern literate societies, as it serves as a foundation for appropriate educational, professional, and social functioning.

Developmental Dyslexia (DD) poses a significant challenge in achieving fluent and accurate reading, despite adequate cognitive abilities and educational opportunities (1). DD is widely acknowledged as one of the most common neurodevelopmental disorders in childhood, significantly affecting the overall well-being and mental health of individuals (2, 3). Furthermore, the adverse effects of DD persist into adulthood, leading to long-term consequences (4).

Extensive research has focused on investigating the neurocognitive architecture of reading, revealing the involvement of a widespread brain network responsible for different reading processes (for a review see 5). This interconnected reading network encompasses three main networks: left dorsal temporo-parietal, left ventral occipito-temporal, and left inferior frontal regions (5). Namely, the left dorsal temporo-parietal regions, which include the posterior superior temporal gyrus, supramarginal gyrus, and angular gyrus, serve as key areas for graphene-phoneme conversion. Within this context, the posterior superior temporal gyrus is implicated in phonological analyses, the supramarginal gyrus connects phonemes to graphemes, and the angular gyrus likely participates in processing word meanings (6). Moreover, the left ventral occipito-temporal regions are suggested to undergo progressive specialization for orthographic coding during literacy acquisition (6). Finally, the left inferior gyrus emerges as crucial for storing sound and sequencing information, playing an essential role in word recognition and decoding (5, 6).

There is consistent evidence demonstrating reduced activation in the left dorsal temporo-parietal and the left dorsal occipito-temporal reading network among children, adolescents and adults with DD (7), which opens up possibilities for the application of brain-directed interventions. In fact, when considering behavioral interventions for DD, effective and long-lasting effects are lacking (8, 9).

Transcranial electrical stimulation (tES) techniques have been proposed as a non-invasive means to target atypical brain functioning in individuals with DD, with the aim of improving specific aspects of reading, such as text accuracy or speed, word recognition, and non-word decoding (6).

tES involves the application of a weak, low-intensity (0.5–2 mA) electrical current through electrodes placed on the scalp over specific cortical areas. These electrodes are typically covered by square or rectangular sponges soaked in saline solution (10). The primary mechanism of action involves modulating neuronal membrane excitability below the threshold for generating action potentials (11, 12).

Among tES techniques, transcranial direct current stimulation (tDCS) is the most widely used method in the pediatric population. It is a polarity-dependent technique, consisting of a direct electrical current delivered via two types of electrodes: anode (positive current) or cathode (negative current) (10). Generally, anodal tDCS induces depolarization of the membrane potential via increasing the excitability of the brain areas, whereas cathodal tDCS induces opposite effects therefore inhibiting cortical excitability (11–13). Neurophysiological studies suggest that tDCS can induce neuroplasticity aftereffects, leading to LTP-like processes through Ca2+ and NMDA receptor-dependent plasticity (11, 14, 15).

Several studies have demonstrated the effects of tDCS, either alone or in combination with reading training, on reading in children and adolescents with DD (16–22). While overall positive outcomes have been observed, the results still show heterogeneity in terms of specific reading aspects improved (e.g., non-word speed vs. text reading accuracy). Over time, different approaches have been employed to address this issue, including the manipulation of electrode montages (e.g., bilateral: left anodal/right cathodal, right anodal/left cathodal; unilateral: left anodal, left cathodal), utilization of different experimental designs (e.g., one-session vs. multi-sessions; between-subjects vs. within-subjects; stand-alone vs. combined with cognitive training), and targeting various brain regions (e.g., inferior frontal gyrus, inferior parietal lobe, posterior middle temporal gyrus, supramarginal gyrus, superior temporal gyrus, temporo-parietal cortex, temporo-parietal junction, and V5/MT). Namely, some studies using different tDCS montages reported similar improvements in the same task [e.g., improvement in text reading was found using several tDCS montages, such as left V5/MT, left/right temporo-parietal junction, left middle temporal gyrus/posterior temporal gyrus, and left/right superior temporal gyrus (23); improvement in nonword reading was found using several tDCS montages, such as left/right temporo-parietal junction, left middle temporal gyrus/posterior temporal gyrus, left/right superior temporal gyrus, and left/right parieto-occipital areas (23)]. Instead, other studies found that the same tDCS montage [e.g., left anodal/right cathodal tDCS over the parieto-temporal regions (16–20) affected different aspects of reading ability (e.g., text, word, and nonword reading)].

Apart from tDCS, transcranial random noise stimulation (tRNS) is another tES technique that is gaining attention, although its application in children and adolescents is still limited (24–27).

tRNS is a polarity-independent technique that involves the application of alternating electrical current at random intensities (e.g., ±0.5 mA) and frequencies (i.e., full spectrum: 0.1–640 Hz; low-frequency range: 0.1–100 Hz; high-frequency range: 100–640 Hz) (28). The exact neurophysiological mechanisms underlying the effects of tRNS are still unclear and subject to debate (29). However, two main hypotheses have been advanced. First, a phenomenon called stochastic resonance has been theorized as responsible for tRNS-induced effects. When an optimal level of noise is added to a weak, subthreshold, noise signal (i.e., brain oscillatory activity), the sum of the signals will exceed the threshold at some point (30). The amplification of subthreshold oscillatory brain activity, which in turn reduces the amount of endogenous noise, improves the signal-to-noise ratio, leading to enhanced perception or cognitive performance. Secondly, *in vitro* and pre-clinical studies suggested that tRNS can induce neuroplasticity processes via LTP through the shortening of hyperpolarization phase and the repetitive re-opening of Na + channels (31–33).

In comparison to tDCS, the effectiveness of tRNS in improving reading abilities has been less extensively explored. A study by Rufener et al. (25) compared the effects of a single session of both tRNS and a different tES technique, transcranial alternating current stimulation (tACS), targeting the bilateral auditory cortex. tACS provides a means to modify cortical excitability, influencing neuroplasticity for the enhancement of cognitive and behavioral processes. Through the application of a weak sinusoidal alternating current, specifically tuned to a particular frequency (Hz), to designated brain regions via scalp electrodes, tACS actively modulates the inherent cortical oscillations. This modulation occurs through the entrainment mechanism, regulating and enhancing brain network communication. Rufener’s study aimed to investigate the online effects of tES on auditory phoneme processing in adolescents (Study 1) and adults (Study 2) with DD. While a positive effect emerged in adolescents only during tACS compared with tRNS and sham condition, a significant improvement was found in adults during tRNS compared with the sham condition. Furthermore, a very recent study by Bertoni et al. (34) found that a single session of active tRNS applied over the bilateral posterior parietal cortex, significantly improved word reading in typical adult readers compared to the sham condition. These findings, although heterogeneous, suggest that tRNS may be a potentially effective method for improving reading and related processes, such as auditory phoneme processing, in individuals with or without DD.

Nevertheless, to the best of our knowledge, the direct effects of tRNS on reading in individuals with DD have not been tested. Additionally, although tDCS and tRNS have been compared in populations with and without neurodevelopmental disorders [e.g., ADHD: (26, 27); healthy adults: (35, 36)], their effects have not been directly compared in individuals with DD. Regarding children and adolescents with ADHD, tRNS has shown to be more effective than tDCS in improving ADHD symptoms, working memory, and processing speed (26, 27). Concerning healthy adults, tRNS has demonstrated better results compared to tDCS in tasks involving working memory, divergent/convergent thinking, and auditory perception (35–37).

Both tDCS and tRNS are considered highly promising tES techniques for the pediatric population due to their favorable safety profile, high tolerability, versatility, ease of use, and low cost (26, 38–43). Interestingly, concerning safety and feasibility, both neurophysiological and behavioral studies [respectively (26, 36, 38)] showed that tRNS has higher skin perception thresholds, lower response rates to adverse events, and more effective blinding, making it a preferable option for the pediatric population.

The current study aims to investigate whether hf-tRNS is effective in improving reading abilities and whether it may outperform tDCS in specific reading aspects. The main goal is to compare the effects of a single online session of hf-tRNS, tDCS, and sham stimulation on text, word, and non-word reading accuracy and speed in children and adolescents with DD. This proof-of-concept study will also assess the safety, tolerability, and blinding parameters of both tES techniques and compare them.

If hf-tRNS proves to be at least as effective as tDCS in improving reading outcomes, these results would provide valuable insights for the development of multi-session hf-tRNS protocols as a potential treatment approach for individuals with DD.

## Methods

### Ethical committee

The local research ethics committee (process number 2639_OPBG_2021) has granted ethical approval for this study, which has been registered on ClinicalTrials.gov (ID: NCT05832060). The study will be conducted in compliance with the Declaration of Helsinki. The protocol adheres to the SPIRIT guidelines (Standard Protocol Items: Recommendations for Interventional Trials) and is prepared using the SPIRIT 2013 Checklist (44). See Supplementary materials S1.

### Participants

Participants will be recruited during the daily clinical activities of the I.Re.Ne Lab (Innovation and Rehabilitation in Neurodevelopment Lab) by neuropsychiatrists, psychologists, and speech therapists from the Child and Adolescent Neuropsychiatry Unit of the Bambino Gesù Children’s Hospital in Rome. The I.Re.Ne Lab offers a diagnostic service for learning disorders, involving a comprehensive psycho-diagnostic assessment. This assessment primarily encompasses the evaluation of intellectual capacity, academic skills, and the emotional-behavioral profile of children and adolescents below 18 years old. When appropriate, participants will be selected from a large database managed by the Head of the Unit (S.V.), which includes several hundred patients evaluated in accordance with the good clinical practices for neurodevelopmental disorders (1). Research assistants will contact selected participants via phone and email to provide information about the ongoing project and assess their interest in participating. All participants and their parents will receive comprehensive instructions regarding the experimental procedures and objectives. No financial compensation or reward will be provided for the participants. The principal investigator will obtain written consent before participants are enrolled in the study. The enrolment started on the 29 August 2023 and will finish on August 2025.

### Clinical eligibility

The inclusion criteria will be as follows: (1) right-handed Italian-native speakers of both genders, who have been diagnosed with DD based on the Diagnostic and Statistical Manual of Mental Disorders, Fifth Edition [DSM-5; (1)] and national recommendations. Their assessment will be conducted by experienced psychologists and neuropsychiatrists using “gold standard” assessment tools (45–48) and considering their developmental history; (2) intelligence quotient (IQ) ≥ 85; (3) age between 8 years and 13 years and 11 months; and (4) normal or corrected-to-normal vision; (5) normal auditory capabilities.

The exclusion criteria will be as follows: (1) presence of another primary psychiatric diagnosis (e.g., depression, anxiety), autism, or ADHD; (2) personal history of neurological/medical/genetic diseases; (3) personal or first-degree relatives’ history of epilepsy; (4) receiving any concomitant treatment for DD during the enrolment in the project; and (5) currently receiving any Central Nervous System (CNS)-active drug treatment.

### Clinical assessment

The assessment will be conducted in an adequately illuminated and quiet room.

Participants’ cognitive level will be evaluated using non-verbal cognitive tests such as the Colored Progressive Matrices [CPM; (49)] and the Standard Progressive Matrices [SPM; (50)]. Alternatively, a multi-componential cognitive test such as the Wechsler Intelligence Scale for Children, 4th edition [WISC-IV; (51)] may be used.

To meet the criteria for DD, participants’ accuracy or speed levels must be at least 1.5 standard deviations (SD) below the normative data for their school-age group, and these difficulties should significantly interfere with their school and daily functioning.

All participants will be assessed for emotional-behavioral symptoms using the Child Behavior Checklist [CBCL; (52)] and the Conners Parent Rating Scale [CPRS; (53)]. The Kiddie Schedule for Affective Disorders and Schizophrenia for DSM-5 (54) will be utilized to exclude neuropsychiatric comorbidities based on the inclusion/exclusion criteria.

Working memory will be assessed using experimental verbal and visual–spatial n-back tasks based on already published procedures (16, 19, 22). Participants will be seated in front of a monitor screen (1920 × 1080) and will be required to indicate if a pronounced alphabetic letter (verbal n-back) corresponds to the last spoken letter or whether a blue-colored box (visual–spatial n-back) moves to the same previous position. If accuracy will achieve 80%, the difficulty will increase and participants will be asked to remember not the last letter spoken or the last position shown, but the second-to-last (2-back), and so on (3-back, 4-back, etc.). In each condition, a total of 35 trials with an interval stimulus of 3.20 s will be presented. An n-back performance index will be calculated and considered for each task (22, 55).

Phonological competences will be evaluated using an auditory-based experimental phoneme blending task based on already published procedures (16, 19, 22). Namely, participants will be instructed to combine individual phoneme sounds to form non-words. Each session will include 10 non-words. The number of accurately blended phonemes (PhonemesAcc) and the response time (in seconds) for each non-word (PhonemesTime) will be recorded and considered for analysis.

Rapid automatized naming will be assessed using a paper-and-pencil experimental Rapid Automatized Naming task (RAN) based on already published procedures (16, 19, 22). In the RAN for letters (RANLetters) or colors (RANColors), participants will be asked to name aloud as quickly and accurately as possible eight letters or eight colored circles presented on a with sheet of A4 paper in lists (56). Total time in seconds will be considered for each task.

To assess attentional abilities, a version (57) of the Posner Cueing Task will be used. Participants will be seated in front of a monitor screen (1920×1080) at eye level and instructed to focus on a central cross. The fixation cross will be accompanied by an either: a left arrow cue, a right arrow cue, or both. The stimulus target “X” will appear a short time later, either to the left or to the right of the fixation cross. Participants will be instructed to maintain fixation on the central cross throughout the trial. In valid trials (120), the target will appear on the side indicated by the arrow, whereas in invalid trials (41), the target will appear on the opposite side of the arrow’s indication. Neutral trials (41) will involve the presence of both arrows, and the target will be randomly presented on one of the sides. Participants will be instructed to react as quickly as possible to the target onset by pressing the “A” key on a computer keyboard, and their reaction times and errors will be recorded.

### Study design

**Figure 1:**
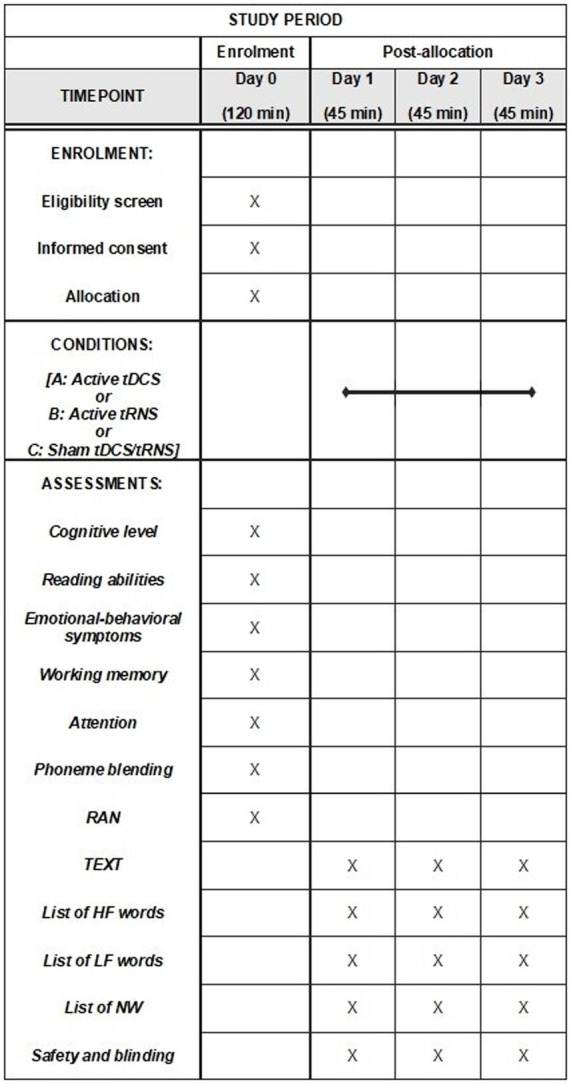
Schedule of enrolment, interventions, and assessment: Recommendations for interventional trials (SPIRIT). tDCS, transcranial direct current stimulation; tRNS, transcranial random noise stimulation; TEXT, texts reading task; HF, high-frequency words reading task; LF, low-frequency words reading task; NW, non-words reading task.

The study will employ a within-subject, randomized, double-blind, and sham-controlled design. Clinical eligibility, including the dyslexia assessment, the neuropsychological assessment, and the psychopathological assessment will be evaluated on Day 0 (Baseline). Participants will be exposed to three conditions (Day 1, Day 2, Day 3): (A) a single active tDCS session; (B) a single active hf-tRNS session; and (C) a single sham tDCS or sham hf-tRNS session. Each condition will be separated from the other by a one-week interval to avoid carry-over effects (27). The ordering of conditions will be counter-matched among participants. After recruitment, participants will be allocated to one of six possible combinations of conditions (i.e., ABC, ACB, BAC, BCA, CBA or CAB). Stratified randomization will be performed by an independent researcher immediately after the participant completes the screening evaluation. This process will ensure assignment masking. Stratified randomization will use the minimal sufficient balancing method to prevent imbalances in baseline characteristics and will take into account participants’ demographics (e.g., age, IQ, and gender) and the severity of DD (calculated as the mean standard deviation between speed and accuracy in the text reading task) (45–48). The randomization information will be retained by an independent researcher until the data collection has been completed (Figure 1).

The principal investigator will dispose of an emergency code for each participant that can be revealed in case of a compelling need, such as a serious adverse event that requires awareness of current interventions to handle the participant’s condition.

Immediately before each session (i.e., Day 1, Day 2, and Day 3) and during the maximum peak of tES effects [approximately 10 min after the start of stimulation; (58), participants will undergo an assessment that will include text, words and non-words reading]. The order of the tasks administered will be randomized and counter-balanced across participants. Each session will last approximately 35 min. This includes 10 min for the reading tasks conducted before the stimulation session, 20 min for the stimulation session (active or sham) combined with concurrent reading tasks (initiated after 10 min of stimulation), and 5 min for the administration of the safety and blinding questionnaire after the stimulation session. In any case, we will take into account the time spent reading the various reading tasks overall (i.e., TEXT, HF, LF, and NW) by each participant. In the event that a participant exceeds 10 min during pre-stimulation reading, we will begin administering the tasks in a way that ensures completion of the reading within the 20-min stimulation period.

**Figure 2:**
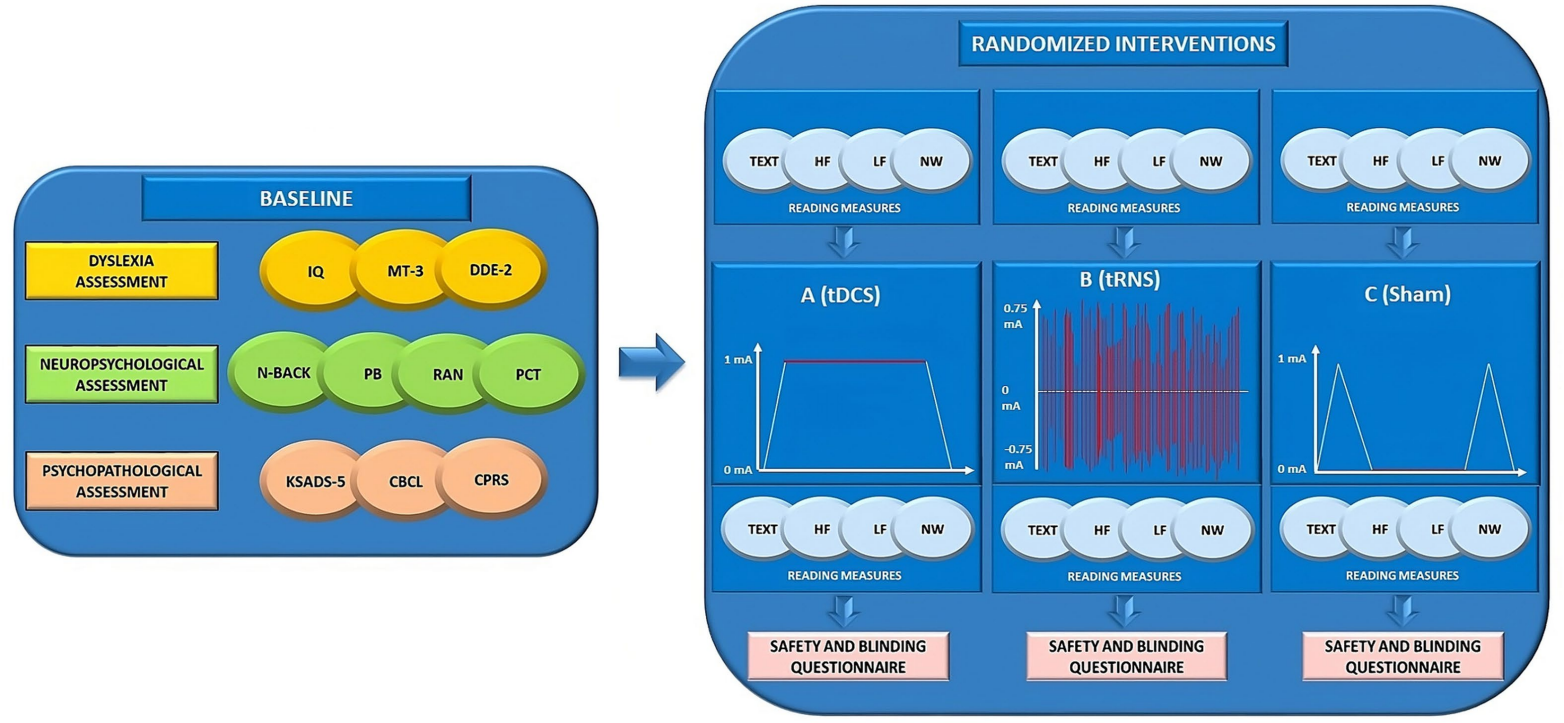
Overview of the study design. IQ, intelligence quotient; MT-3 (46, 47); DDE-2 (45); N-BACK (22, 55); PB, Phoneme Blending; RAN, Rapid Automatized Naming Task; PCT, Posner Cueing Task (57); KSADS-5, Kiddie Schedule for Affective Disorders and Schizophrenia for DSM-5 (54); CBCL, Child Behavior Checklist (52); CPRS, Conners Parent Rating Scale (53); TEXT, texts reading task; HF, high-frequency words reading task; LF, low-frequency words reading task; NW, non-words reading task; tDCS, transcranial direct current stimulation; tRNS, transcranial random noise stimulation; mA, milliamperes.

At the end of the final session (Day 3), participants will be informed of their assigned condition (i.e., ABC, ACB, BAC, BCA, CBA or CAB) and provided with brief feedback on the results that emerged from each session (Figure 2).

### Interventions

To ensure protocol adherence and minimize variability, the experimenter responsible for delivering the interventions will complete a structured checklist before each stimulation session. This checklist will include participants’ information, details of the procedures to be applied, and dose parameters. The checklist is adapted from Antal et al. (59). See Supplementary materials S2.

### Transcranial direct current stimulation (tDCS)

According to the International 10–20 System, the anodal electrode will be placed over the left temporo-parietal region (TP7/P7), while the cathodal electrode will be placed over the contralateral temporo-parietal region (TP8/P8). Consistent with our previous tDCS protocols in developmental age (16–22), in the active condition a constant current at 1 mA intensity will be delivered for 20 min, with a density of 0.04 mA/cm^2^, preceded by 30 s of rump up (0 mA to 1 mA) and succeeded by 30 s of rump down (1 mA to 0 mA). Direct current will be delivered by a battery driven, direct current stimulator (BrainStim stimulation by E.M.S. s.r.l.— Bologna, Italy) via a pair of identical, circular (25 cm^2^) saline-soaked (0.90 mol) sponge electrodes. tDCS parameters are based on already published procedures (16–22).

### Transcranial random noise stimulation (tRNS)

During the active hf-tRNS session, participants will receive 0.75 mA (±0.375 mA) of tRNS (100–500 Hz) to their temporo-parietal regions via 2 saline-soaked (0.90 mol) 25 cm^2^ circular sponges, placed over TP7/P7 and TP8/P8 based on the International 10–20 System. The current will be delivered by a BrainStim stimulator (E.M.S. s.r.l.; Bologna, Italy) for 20 min per session, as in previous tRNS protocols (24, 26, 60–64). The impedance of the electrodes will be checked before and during the application of hf-tRNS to ensure that it remains below 10 kW. tRNS parameters are based on already published procedures (24, 55).

### Sham tES conditions

To control for potential placebo effects, participants during the sham condition will undergo the same procedures as those in the active conditions (active tDCS, active hf-tRNS). This includes using the same electrode positioning and tRNS/tDCS equipment turn-on time (30 s). Apart from this short stimulation, during the sham condition participants will not receive the real stimulation (0 mA) for the rest of the session.

Among all participants, half of them will undergo a sham tDCS session (with the same electrode positioning and tDCS equipment turn-on time), while the other half will undergo a sham tRNS session (with the same electrode positioning and tRNS equipment turn-on time).

All participants, their families and the evaluators will be blinded to the stimulation conditions.

### Reading tasks

Four different reading tasks (text – TEXT; high-frequency words – HF; low-frequency words – LF; non-words – NW) will be presented to the participants in the Italian language. Participants will be required to read aloud as quickly and accurately as possible.

TEXT derived from an Italian novel (65). Items in HF list and LF list were matched for Italian written word frequency, number of letters and syllables, bigram frequency (according to CoLFIS)^1^ and mean onset reaction time. To avoid the repetition effect, multiple versions of each reading task will be administered. This will include different versions presented at baseline (before stimulation) and during each stimulation session, resulting in a total of six versions across the study. The purpose of this approach is to minimize the impact of task familiarity. To control for the influence of fatigue, the order of the reading tasks will be counterbalanced between the three conditions (tDCS, hf-tRNS, and sham).

Regarding reading task characteristics, each TEXT will be composed of approximately 400 syllables, each list of HF will be composed of 30 high frequency words (over 70 syllables long), each list of LF will be composed of 30 low frequency words (over 70 syllables long), and each list of NW will be composed of 30 non-words (over 70 syllables long).

For each reading task (TEXT, HF, LF, and NW), both reading speed and accuracy will be measured and considered. Concerning TEXT reading accuracy, an error point will be assigned in presence of substitution, omission, and/or addition of syllables. A 0.5 error will be assigned in case of auto-correction during reading. The number of words correctly read will be considered, and the percentage of accuracy will be calculated via dividing the number of correctly read stimuli by the total number of stimuli presented and multiplying the result by 100. For the remaining tasks (HF, LF, NW), an error point will be assigned in presence of substitution, omission, and/or addition of syllables, while auto-corrections during reading will not be treated as errors. The number of errors will be considered, and the percentage of errors will be calculated via dividing the number of errors by the total number of stimuli presented and multiplying the result by 100.

Concerning reading speed, syllables per seconds (syll/s) will be considered by dividing the total number of pronounced syllables by the time taken to complete the reading task (in seconds) for all tasks (TEXT, HF, LF, NW).

To ensure that the six versions of each reading task (TEXT, HF, LF, and NW) are comparable in terms of difficulty for both accuracy and speed, a pilot study was conducted with a group of typically developing readers. This preliminary investigation helped determine that the task versions will be appropriately matched in difficulty across the different conditions (see Supplementary materials S3).

The primary outcome of the study will be the changes in TEXT reading accuracy during the active tDCS and active hf-tRNS sessions compared to the sham session (tDCS/tRNS), in comparison to the baseline measurement on Day 0. The primary outcome has been defined based on the results of Costanzo et al. (16), whereby a single session of active left anodal/right cathodal tDCS over temporo-parietal regions led to significant improvements on TEXT reading accuracy compared to control conditions.

Similarly, the secondary outcomes of the study will examine changes in TEXT reading speed, as well as changes in the accuracy and speed of the other reading tasks (HF, LF, and NW) during the active tDCS and active hf-tRNS sessions compared to the sham session (tDCS/tRNS), in comparison to the baseline measurement on Day 0.

### Safety, tolerability, and blinding assessment

Adverse effects will be assessed using a questionnaire adapted from Antal et al. (59). Participants will be asked to complete the questionnaire after each session. The questionnaire includes items related to potential adverse effects such as headache, tingling, skin redness, neck pain, scalp pain, itching, drowsiness, difficulty concentrating, burning sensation, and acute mood change. In addition, other information regarding when the adverse event occurred, its duration, and where the discomfort is located will be recorded. Participants will quantify the severity of adverse effects as follows: (0) absent; (1) mild; (2) moderate; and (3) severe.

The questionnaire will also inquire about participants’ perception of whether they received active stimulation or not. For more details, see Supplementary materials S4.

### Protection of risks

To reduce any risks associated with tDCS/tRNS, participants will be closely monitored throughout the stimulation sessions and encouraged to report any discomfort. Should they experience uncomfortable scalp sensations or headaches, the stimulation will be immediately halted. All tDCS/tRNS sessions will be conducted and supervised continuously by a trained experimenter.

### Sample size

The sample size is calculated on the primary outcome by *a priori* analysis in G*Power, version 3.1.9.7 (The G*Power Team, Düsseldorf, Germany). Based on the study design and assumptions, the estimated results suggest that participants who receive a single session of active tDCS or hf-tRNS will demonstrate an improvement in TEXT reading accuracy compared to their baseline performance. On the other hand, participants who receive a single session of sham tDCS or sham tRNS are not expected to show a significant change in their performance compared to baseline. While the design of this project has never been employed in DD, we will refer to (i) a previous study by Costanzo and collaborators (16) who prove the effectiveness of a one-session active left anodal/right cathodal tDCS over temporo-parietal regions in a within-subject design; (ii) a study with similar features (e.g., one-session, sham-controlled experiment, comparing baseline vs. tDCS vs. tRNS vs. sham) assessing other cognitive functions (35).

Based on these previous results, to be cautious and conservative, we estimate a medium effect size (f) of 0.25. With an estimated *f* = 0.25, α value = 0.05 (i.e., probability of false positives of 5%), and *β* = 0.80 (i.e., at least 80% power), the sample size is 24 as calculated using a Repeated Measures-Analysis of Variance (RM-ANOVA) model with four within factors (i.e., baseline, active tDCS, active hf-tRNS, and sham).

### Statistical analyses and expected results

The Shapiro–Wilk test will be used to test the normality of the data and Levene’s test for the homogeneity of variances. When data will be normally distributed and the assumption of homogeneity will not be violated, parametric analyses will be computed. When one assumption will not be met, non-parametric tests will be conducted or a log-transformation of the distribution will be applied. Sphericity will be verified by Mauchly’s sphericity test and when not met, Greenhouse–Geisser correction will be applied.

Chi-Square analyses will be used to compare the groups on demographic and safety and blinding measures (categorical variables).

Covarying for age, a preliminary analysis to test the effect of the four repetitions (Day 0, Day 1, Day 2, and Day 3) of the reading tasks (accuracy and speed of TEXT, HF, LF, and NW) will be conducted.

RM-ANOVA will be used to compare reading measures (TEXT, HF, LF, and NW accuracy and speed), separately, with conditions (Day 0, A, B, C) as a within-subjects factor and age as covariate.

*Post hoc* comparisons will be assessed using Tukey’s honest significance test.

Partial eta squares (η_p_^2^) will be used as measures of effect sizes.

We hypothesize that:

- hf-tRNS will be at least as effective as tDCS in improving TEXT reading accuracy compared with sham condition (primary outcome);
- hf-tRNS and tDCS will improve performance in the remaining reading tasks (TEXT reading speed and HF, LF, and NW reading accuracy and speed) compared with sham condition;
- hf-tRNS and tDCS will be as safe as sham condition (i.e., neither tDCS nor hf-tRNS will induce severe AEs or lead to dropouts due to intolerance to stimulation);
- the blinding will be kept across the conditions.

### Documentation, monitoring, and data management

The OPBG Contract Research Organization is responsible for designing the Case Report Form (CRF) and other data collection modules. The principal investigator must store all data for each subject. If any required data are missing, an explanation should be provided on the appropriate data collection forms and transcribed on the CRF page if necessary.

In case of clarifications, the investigator must respond within agreed timelines. Researchers will organize the retention of patient identification codes for a period of at least 15 years. Patient records must be retained for a minimum of 15 years.

The principal investigator will be the data custodian and will store the data at the Child and Adolescent Neuropsychiatry Unit. The subject data will be anonymized and coded. Paper records will be kept in a locked drawer, with the key held by the principal investigator. The database containing the coded data will have protected access, with the key also held by the principal investigator.

The investigator will allow monitoring, verification, review by the Institutional Review Board/Independent Ethics Committee, and inspection by regulatory authorities related to the study, providing direct access to original Data/Documents. The CRF and original documents can be consulted and verified by the clinical monitor and inspected by regulatory authorities at any time.

For all data that cannot be printed, their transcription on the patient’s study data collection folder will serve as the original record of such data. The obtained results will be provided to the participants and their parents or guardians at the conclusion of the experiment.

### Withdrawal of subjects and intervention modifications

Any suspensions or interruptions of the treatment will be documented by the investigator. Subjects will be immediately withdrawn from the study if there are foreseeable or unforeseeable adverse effects. If withdrawal occurs during the intervention phase, the subject’s data will not be considered for analysis. Replaced subjects will be those who undergo the complete protocol.

### Early conclusion or suspension of the study

The promotor may terminate the study at any time and promptly notify the investigators and ethics committee. Patients will be examined as soon as possible and will continue to be followed according to normal clinical practice.

### Definition of study conclusion

The study is expected to conclude by August 2025. The last patient may be enrolled by July 2024. Investigators must not implement any deviation or modification of the Clinical Trial Protocol without prior favorable opinion from the ethics committee.

## Discussion

Over the last decades, several interventions have been developed to improve reading skills in children and adolescents with DD (8, 9), although with poor results in term of efficacy and long-term achievements. The growing understanding of the neurobiology of reading in individuals with and without DD has led to the exploration of brain-directed methods as a potential new frontier in the treatment of DD.

Promising findings on the application of tES to improve reading in individuals with DD are already available and represent the rationale for this project (23). However, there is still a need to bridge the gap and reduce the variability in results, thereby optimizing neuromodulation protocols. This includes identifying the most effective technique to employ.

The present study aims to compare two tES techniques already used in pediatrics, namely tDCS and hf-tRNS, to investigate (i) which is more effective in improving reading abilities in children and adolescents with DD, and (ii) which is the most suitable in terms of safety and tolerability.

tDCS is definitely the most widely used technique to ameliorate reading performance in DD. One of the reasons is that numerous investigations have allowed to determine reliable tDCS parameters in terms of intensity and duration to achieve plastic after-effects, particularly by combining tDCS with transcranial magnetic stimulation at the level of the motor cortex (28). Moreover, tDCS polarity-dependent nature resulted suitable for left-hemisphere lateralization of reading [i.e., hypoactivation of a left hemisphere brain network and hyperactivation of contralateral homologous regions; for a review, see (6)], considering the possibility to simultaneously manipulate excitation/inhibition balance, via anodal/cathodal montage over target brain areas. Consistently, we will employ the left anodal/right cathodal montage accordingly to several studies that demonstrated the superiority of this montage in improving reading performance (23). Specifically, we will target the temporo-parietal regions that are crucially involved in reading, particularly in the process of letter-to-sound conversion (66–68).

Conversely, tRNS is a polarity-independent form of tES that employs the same electrode arrangement as tDCS to boost neuronal activity, but uses both electrodes to increase cortical excitability (28, 31). Previous findings have shown stronger effects of tRNS compared to tDCS on language and learning abilities, including mathematical skills (69), as well as on other cognitive (26, 27, 35, 36) and perceptual processes (39).

Our selection of tES parameters, including intensity, density, duration, and frequency, will be based on previous research demonstrating the safety and beneficial effects of these parameters on reading performance (16–22) and cognitive tasks (24, 60, 63, 70, 71).

Concerning tDCS, we will employ a tDCS set-up that delivers a constant current at 1 mA intensity for 20 min. This configuration aligns with previous studies that have demonstrated the safety and tolerability of this approach in pediatric populations (16–22).

As for hf-tRNS, we will apply a current intensity of 75% of 1 mA. The choice to administer a 75% of 1 mA current was reached considering the parameters that affect the distribution and concentration of current at the stimulation site. These factors include a reduced thickness of the scalp, decreased cerebrospinal fluid volume, and the comparatively smaller head dimensions in the pediatric population (72–74). Concerning the frequency band, tRNS can administer current in three distinct frequency ranges: a full-frequency range (typically spanning from 0.1 to 640 Hz), a low-frequency range (typically ranging from 0.1 to 100 Hz), and a high-frequency range (typically ranging from 101 to 640 Hz). Our decision to use the hf-tRNS set-up is based on evidence demonstrating that only at 100–500 Hz of frequency tRNS provides consistent and long-lasting cortical excitability (31).

With respect to the experimental design, the choice of a within-subjects design is motivated by the aim to reduce inter-subject variability, which is well-known in the effects of tES [for a review, see (75)]. Within-subjects designs offer comparable accuracy to between-subjects designs and enhance statistical power even with a smaller number of participants (76–78) and suppressing inter-subject variability (79, 80). In between-subject designs, there is a greater risk that stable factors specific to each participant may influence responses to tES conditions. In particular, it has been demonstrated that individuals anatomical differences (e.g., skull thickness, cortex morphology, and gyrification) could influence the electric field reaching the brain (74, 81, 82), and consequently modulate the behavioral effects of tES. Moreover, several studies showed that individual’s genotype seems to modulate synaptic plasticity and neurotransmitters expression, somehow affecting the effects of tES (83). Furthermore, it has been found that major demographic characteristics such as gender (84, 85) and age (83–85) could contribute to the inter-subject variability in the effects of tES.

Among the main purposes of the present study is to compare the safety and tolerability profile of tDCS and tRNS in pediatrics, in order to verify which intervention is safer and more suitable for use as standard-of-care intervention for children and adolescents with DD. While tES has been increasingly applied in developmental age, there are limited studies that have systematically investigated the safety and tolerability of tES in children and adolescents [(40, 86–88); for a review, see (41)], overall promoting tES as a safe method as for adults. Furthermore, while evidence suggests that tRNS is a safe and potentially less perceptible technique compared to tDCS (26, 36, 38), only one study has directly compared the safety and tolerability of the two techniques in children and adolescents (26), demonstrating that tRNS is less perceptible and potentially preferable in pediatrics. In particular, Berger et al. (26) documented a lower incidence of participants reporting adverse events with tRNS compared to tDCS (6 participants vs. 13 participants, respectively), particularly concerning the symptom “itching” (7% of sessions vs. 13% of sessions, respectively). Similarly, Pena and colleagues also observed a higher overall occurrence of minor adverse events in the tDCS group compared to the tRNS group. These initial findings align with prior studies by Ambrus and colleagues (38), who suggested in their conclusions that future research should explore alternative methods to the sham condition (such as placebo itching) to ensure better blinding integrity.

### Limitations

While emphasizing the novelty of our approach and its rationale, we would also like to discuss some potential limitations.

First, to reduce the number of stimulation sessions per participant and streamline the study, we will not administer a sham session for both tDCS and hf-tRNS. In fact, half of the participants will receive a sham tDCS session, while the other half will receive a sham hf-tRNS session. However, we consider this limitation not to pose a significant bias, as both sham conditions entail identical placebo stimulation.

Second, the absence of neurophysiological measures, such as EEG, represent a limitation of the study. These measures could provide valuable insights into the underlying neurophysiological mechanisms of tDCS and hf-tRNS and help interpret the observed behavioral outcomes. Given the partial understanding of the neurophysiological effects of tES techniques, incorporating neurophysiological measures alongside behavioral assessments would be beneficial for future studies.

Finally, future studies should include a longitudinal monitoring of the effects of tES on reading abilities, with the aim of assessing the clinical relevance of experimental results.

## Conclusion

In conclusion, the present study could provide new insights regarding the effectiveness of a newly-wave tES method never used in children and adolescents with DD, the hf-tRNS, potentially paving the way for further studies involving multiple hf-tRNS sessions. Considering the absence of studies evaluating the efficacy of hf-tRNS in improving reading skills and comparing it with the effects obtained with tDCS, we preferred to be cautious in advancing hypotheses of superiority of one technique over the other. However, while expecting comparable effects of the two techniques on outcome measures, we believe that this study may help optimize neuromodulation protocols for dyslexia in the future, for example, by considering the magnitude of the effects obtained by the two techniques. Of importance, this project will employ an experimental design that keeps the study burden for DD participants at a minimum, while providing key steps to understand which tES technique may be most effective for multi-session treatment applications.

The opportunity to compare tDCS and hf-tRNS in the same experimental setting, investigating whether differ in modulating reading performance and in terms of safety and tolerability, will be a valuable step forward to identify brain-directed treatments for DD. Instead, it will be crucial to assess whether the potential comparability on behavioral outcomes will go hand in hand with comparability on tolerability and safety parameters.

## Ethics statement

The study was approved by Bambino Gesù Children’s Hospital ethics committee (process number 2639_OPBG_2021). The study was conducted in accordance with the local legislation and institutional requirements. Written informed consent for participation in this study was provided by the participants’ legal guardians/next of kin.

## Author contributions

AB: Conceptualization, Writing – original draft, Writing – review & editing. GL: Writing – original draft, Writing – review & editing. CV: Writing – review & editing. SV: Conceptualization, Supervision, Writing – review & editing. DM: Conceptualization, Supervision, Writing – review & editing.

## Footnotes

^1^ https://linguistica.sns.it/CoLFIS/Home.htm
