# Supplementary material for: Effects of online tDCS and hf-tRNS on reading performance in children and adolescents with developmental dyslexia: a study protocol for a cross sectional, within-subject, randomized, double-blind, and sham-controlled trial
Source: Front Neurol. 2024 Mar 12;15:1338430. doi: 10.3389/fneur.2024.1338430 (PMC10964771; doi:10.3389/fneur.2024.1338430)
Supplement: Supplementary file 1 [file Data_Sheet_1.pdf]

Supplementary Materials

Effects of online tDCS and hf-tRNS on reading performance in children and adolescents with developmental dyslexia: a study protocol for a cross-sectional, within-subject, randomized, double-blind, and sham-controlled trial

S1. SPIRIT 2013 Checklist

SPIRIT 2013 Checklist: Recommended items to address in a clinical trial protocol and related documents*

Section/item	Item No	Description	Addressed on page number
**Administrative information**			
Title	1	Descriptive title identifying the study design, population, interventions, and, if applicable, trial acronym	________
Trial registration	2a	Trial identifier and registry name. If not yet registered, name of intended registry	________
	2b	All items from the World Health Organization Trial Registration Data Set	na___________
Protocol version	3	Date and version identifier	na___________
Funding	4	Sources and types of financial, material, and other support	_______
Roles and responsibilities	5a	Names, affiliations, and roles of protocol contributors	____
	5b	Name and contact information for the trial sponsor	na___________
	5c	Role of study sponsor and funders, if any, in study design; collection, management, analysis, and interpretation of data; writing of the report; and the decision to submit the report for publication, including whether they will have ultimate authority over any of these activities	na___________
	5d	Composition, roles, and responsibilities of the coordinating centre, steering committee, endpoint adjudication committee, data management team, and other individuals or groups overseeing the trial, if applicable (see Item 21a for data monitoring committee)	na___________
**Introduction**			

Background and rationale	6a	Description of research question and justification for undertaking the trial, including summary of relevant studies (published and unpublished) examining benefits and harms for each intervention	_____
	6b	Explanation for choice of comparators	_____
Objectives	7	Specific objectives or hypotheses	________
Trial design	8	Description of trial design including type of trial (eg, parallel group, crossover, factorial, single group), allocation ratio, and framework (eg, superiority, equivalence, noninferiority, exploratory)	____
**Methods: Participants, interventions, and outcomes**			
Study setting	9	Description of study settings (eg, community clinic, academic hospital) and list of countries where data will be collected. Reference to where list of study sites can be obtained	_________
Eligibility criteria	10	Inclusion and exclusion criteria for participants. If applicable, eligibility criteria for study centres and individuals who will perform the interventions (eg, surgeons, psychotherapists)	______
Interventions	11a	Interventions for each group with sufficient detail to allow replication, including how and when they will be administered	_____
	11b	Criteria for discontinuing or modifying allocated interventions for a given trial participant (eg, drug dose change in response to harms, participant request, or improving/worsening disease)	________
	11c	Strategies to improve adherence to intervention protocols, and any procedures for monitoring adherence (eg, drug tablet return, laboratory tests)	na____________
	11d	Relevant concomitant care and interventions that are permitted or prohibited during the trial	______
Outcomes	12	Primary, secondary, and other outcomes, including the specific measurement variable (eg, systolic blood pressure), analysis metric (eg, change from baseline, final value, time to event), method of aggregation (eg, median, proportion), and time point for each outcome. Explanation of the clinical relevance of chosen efficacy and harm outcomes is strongly recommended	____
Participant timeline	13	Time schedule of enrolment, interventions (including any run-ins and washouts), assessments, and visits for participants. A schematic diagram is highly recommended (see Figure)	___

Sample size	14	Estimated number of participants needed to achieve study objectives and how it was determined, including clinical and statistical assumptions supporting any sample size calculations	_______
Recruitment	15	Strategies for achieving adequate participant enrolment to reach target sample size	na____________

Methods: Assignment of interventions (for controlled trials)

Allocation:

Sequence generation	16a	Method of generating the allocation sequence (eg, computer-generated random numbers), and list of any factors for stratification. To reduce predictability of a random sequence, details of any planned restriction (eg, blocking) should be provided in a separate document that is unavailable to those who enrol participants or assign interventions	______
Allocation concealment mechanism	16b	Mechanism of implementing the allocation sequence (eg, central telephone; sequentially numbered, opaque, sealed envelopes), describing any steps to conceal the sequence until interventions are assigned	______
Implementation	16c	Who will generate the allocation sequence, who will enrol participants, and who will assign participants to interventions	______
Blinding (masking)	17a	Who will be blinded after assignment to interventions (eg, trial participants, care providers, outcome assessors, data analysts), and how	______
	17b	If blinded, circumstances under which unblinding is permissible, and procedure for revealing a participant's allocated intervention during the trial	______

Methods: Data collection, management, and analysis

Data collection methods	18a	Plans for assessment and collection of outcome, baseline, and other trial data, including any related processes to promote data quality (eg, duplicate measurements, training of assessors) and a description of study instruments (eg, questionnaires, laboratory tests) along with their reliability and validity, if known. Reference to where data collection forms can be found, if not in the protocol	______
	18b	Plans to promote participant retention and complete follow-up, including list of any outcome data to be collected for participants who discontinue or deviate from intervention protocols	na____________

Data management	19	Plans for data entry, coding, security, and storage, including any related processes to promote data quality (eg, double data entry; range checks for data values). Reference to where details of data management procedures can be found, if not in the protocol	______
Statistical methods	20a	Statistical methods for analysing primary and secondary outcomes. Reference to where other details of the statistical analysis plan can be found, if not in the protocol	______
	20b	Methods for any additional analyses (eg, subgroup and adjusted analyses)	______
	20c	Definition of analysis population relating to protocol non-adherence (eg, as randomised analysis), and any statistical methods to handle missing data (eg, multiple imputation)	na________
**Methods: Monitoring**			
Data monitoring	21a	Composition of data monitoring committee (DMC); summary of its role and reporting structure; statement of whether it is independent from the sponsor and competing interests; and reference to where further details about its charter can be found, if not in the protocol. Alternatively, an explanation of why a DMC is not needed	______
	21b	Description of any interim analyses and stopping guidelines, including who will have access to these interim results and make the final decision to terminate the trial	______
Harms	22	Plans for collecting, assessing, reporting, and managing solicited and spontaneously reported adverse events and other unintended effects of trial interventions or trial conduct	______
Auditing	23	Frequency and procedures for auditing trial conduct, if any, and whether the process will be independent from investigators and the sponsor	na____________
**Ethics and dissemination**			
Research ethics approval	24	Plans for seeking research ethics committee/institutional review board (REC/IRB) approval	na____________
Protocol amendments	25	Plans for communicating important protocol modifications (eg, changes to eligibility criteria, outcomes, analyses) to relevant parties (eg, investigators, REC/IRBs, trial participants, trial registries, journals, regulators)	______
Consent or assent	26a	Who will obtain informed consent or assent from potential trial participants or authorised surrogates, and how (see Item 32)	______

	26b	Additional consent provisions for collection and use of participant data and biological specimens in ancillary studies, if applicable	na___________
Confidentiality	27	How personal information about potential and enrolled participants will be collected, shared, and maintained in order to protect confidentiality before, during, and after the trial	______
Declaration of interests	28	Financial and other competing interests for principal investigators for the overall trial and each study site	______
Access to data	29	Statement of who will have access to the final trial dataset, and disclosure of contractual agreements that limit such access for investigators	______
Ancillary and post-trial care	30	Provisions, if any, for ancillary and post-trial care, and for compensation to those who suffer harm from trial participation	na___________
Dissemination policy	31a	Plans for investigators and sponsor to communicate trial results to participants, healthcare professionals, the public, and other relevant groups (eg, via publication, reporting in results databases, or other data sharing arrangements), including any publication restrictions	na______
	31b	Authorship eligibility guidelines and any intended use of professional writers	na___________
	31c	Plans, if any, for granting public access to the full protocol, participant-level dataset, and statistical code	na___________
**Appendices**			
Informed consent materials	32	Model consent form and other related documentation given to participants and authorised surrogates	na___________
Biological specimens	33	Plans for collection, laboratory evaluation, and storage of biological specimens for genetic or molecular analysis in the current trial and for future use in ancillary studies, if applicable	na___________

*It is strongly recommended that this checklist be read in conjunction with the SPIRIT 2013 Explanation & Elaboration for important clarification on the items. Amendments to the protocol should be tracked and dated. The SPIRIT checklist is copyrighted by the SPIRIT Group under the Creative Commons "Attribution-NonCommercial-NoDerivs 3.0 Unported" license.

S2. Checklist adapted from Antal et al. (2017)

A structured checklist increases the reproducibility of studies, minimizes deviations from a given protocol and diminishes variability. A structured checklist is thus the recommended procedure for enhancing reliability and comparability in publications of TES experiments/trials.

1) Participant information

- Age:
- Gender:
- Handedness:
- Head size (distance in cm: inion – nasion, ear to ear distance):
- *Previous experience with TES (additional information of potential relevance):*
- Medication (Depending on the type of study an even more precise documentation may be necessary, measurement of drug levels may be considered), label and dose:

  Within last hours

  Within last days

  Within last months
- Caffeine consumption (cups) (indicate the best currently relevant estimate):

  Within last 12 h

  Average within last months
- Nicotine consumption (cigarettes per day) (indicate the best currently relevant estimate):

  Within last 4 h (*half life of Nicotine: 2 h*)

  Within last 48 h (*half life metabolite cotinine: 10–37 h*)
- Alcohol consumption (drinks) (indicate the best currently relevant estimate):

Within last 24 h

Average with last months (how many months?)

- Drugs (e.g. marijuana) consumption (to be specified):

(for comparability important that unit is given and comparable measures are noted)

- Hormonal/menstrual cycle of female subjects
- *In case of patients non-neuropsychiatric comorbidities:*

2) Procedures applied, Dose parameters *(sufficient information about the stimulation parameters should be provided in order to replicate or model the stimulation dose independently based on these parameters)*

- Type of stimulation (complicated waveforms with drawings):
- Metric to be used (e.g., behavioral, cognitive, EEG, MEP, MRI):
- Product number and model of stimulator used (consider Nr. as encoded in case of multiple stimulators available):
- Stimulation intensity (peak-to-baseline):
- Stimulation duration:

  Duration of ramping

  Fragmented stimulation (interval duration)

- Type and number of electrodes:
- Electrode positions:
- Electrode polarities in case of tDCS:
- Position of cable fixation at electrode:
- Electrode shape:

  target electrode:

return electrode:

- Electrode size:

target electrode:

return electrode:

- Electrode impedance:

target electrode:

return electrode:

- Method of allocation of electrode position (neuronavigation, MEP hot spot, modeling etc.):
- Electrode-skin interface (any skin preparation steps):
- Type of fixation:

saline (molarity?), in case of cream, brand:

Other factors to be considered

- Tasks/status during stimulation (if any): o Not specified or regulated

Specified/regulated: details __

- Day time of the experiment (from – to):
- Attention (level of arousal)

1. before stimulation:
2. during stimulation (optimal results expected with relaxation, not during arousal or sleepiness):
3. after stimulation:
4. Number of hours in sleep during the last night:

- Prior motor activity (i.e. cycling before stimulation, if yes, please define the duration):
- Prior rest (sleep) before stimulation:

- Duration of the whole experiment including preparation:

- Number of years in education (of interest in special, e.g. in cognitive studies):

- Additional comments

S3. Pilot study of the reading tasks

A behavioural pre-test was administered to 20 typically developing readers (10 children and adolescents: 5 females; M = 12.03 yrs, SD = 1.38 yrs; 10 young adults: 9 females; M = 27.10 yrs, SD = 2.38 yrs). Each participant had to read aloud as rapid and accurate as possible the following reading tasks:

-10 texts of approximately 400 syllables in length (TEXT);

-9 lists of 30 high frequency words (HF – over 70 syllables long);

-9 lists of 30 low frequency words (LF – over 70 syllables long);

-9 lists of 30 non-words (NW – over 70 syllables long) created by rearranging the character string of real word items.

TEXT was written with Century Schoolbook font, size 13, single-spaced, on a white sheet of A4 paper. HF, LF, NW were arranged in 30-items columns, written with Century Schoolbook font, size 13, single-spaced, on a white sheet of A4 paper. TEXT derived from an Italian novel (Calvino, 1963). Items in HF list and LF list were matched for Italian written word frequency, number of letters and syllables, bigram frequency (according to CoLFIS, http://www.Istc.cnr.it/material/database/colfis) and mean onset reaction time (Barca et al., 2002).

For each of the four reading tasks (TEXT, HF, LF, and NW), reading accuracy and speed data were collected.

Concerning TEXT reading accuracy, an error point was assigned in presence of substitution, omission, and/or addition of syllables. A 0.5 error was assigned in case of auto-correction during reading. The number of words correctly read was considered, and the percentage of accuracy was calculated via dividing the number of correctly read stimuli by the total number of stimuli presented and multiplying the result by 100. For the remaining tasks (HF, LF, NW), an error point was assigned in presence of substitution, omission, and/or addition of syllables, while auto-corrections during reading was not treated as errors. The number of errors was considered, and the percentage of errors

was calculated via dividing the number of errors by the total number of stimuli presented and multiplying the result by 100.

Concerning reading speed, syllables per seconds (syll/s) was considered by dividing the total number of pronounced syllables by the time taken to complete the reading task (in seconds) for all tasks (TEXT, HF, LF, NW).

S3.1. Statistical Analysis

To compare accuracy (percentage of accuracy), analyses of covariance (ANCOVAs) were run for each reading measure (TEXT, HF, LF, and NW) with number of stimuli (10 for TEXT; 9 for HF, 9 for LF, and 9 for NW) as independent factor. The potential effects of age were taken into account by including the participants' age at baseline as a covariate.

The same analyses were used to compare each reading measure on reading speed (syll/sec).

Post-hoc comparisons were run using Tukey's honest significance test. Partial eta square (η_p^2) was used as a measure of effect size.

S3.2. Results

Table S1 shows Means (SDs) of reading accuracy (percentage of accuracy) and speed (syll/sec) for each version of the set of stimuli (TEXT, HF, LF, and NW).

ANCOVA results:

- 10 TEXT [Accuracy: $F(9, 162) = 1.64$, $p = 0.11$, $\eta_p^2 = 0.08$; Speed: $F(9, 162) = 1.40$, $p = 0.19$, $\eta_p^2 = 0.07$];
- 9 lists of HF [Accuracy: not possible to perform due to the absence of minimum variance in the data; Speed: $F(8, 144) = 1.30$, $p = 0.25$, $\eta_p^2 = 0.07$];
- 9 lists of LF [Accuracy: $F(8, 144) = 2.81$, $p = 0.006$, $\eta_p^2 = 0.13$; Speed: $F(8, 144) = 2.44$, $p = 0.017$, $\eta_p^2 = 0.12$]. When considering accuracy, post hoc analysis showed no significant results when comparing the different versions of LF [p always > 0.05]; when considering speed, post hoc analysis

showed the following significant differences: LF_1 > LF_5 ($p = 0.0013$); LF_1 > LF_9 ($p = 0.021$); LF_2 > LF_4 ($p = 0.016$); LF_2 > LF_5 ($p < 0.0001$); LF_2 > LF_7 ($p = 0.007$); LF_2 > LF_9 ($p = 0.0002$); LF_3 > LF_5 ($p = 0.026$).

- 9 lists of NW [Accuracy: $F(8, 144) = 0.67$, $p = 0.72$, $\eta_p^2 = 0.04$; Speed: $F(8, 144) = 1.01$, $p = 0.43$, $\eta_p^2 = 0.05$].

S3.3. Conclusions

For LF exclusively, ANCOVA revealed significance in both accuracy and speed. Given the non-significant results of post-hoc analyses concerning accuracy, we chose to discard versions based solely on post-hoc analyses of speed. As a result, we excluded lists LF_1 and LF_2.

At the end of the selection process, we rerun the same analysis to confirm the non-significance of the ANCOVA.

The accuracy (percentage of accuracy) and reading speed (syll/sec) of each set of stimuli were compared covarying for age, and the following equivalent stimuli were selected (see Table S1 for means and SDs):

- out of 9 versions, 7 lists of LF (LF_3, LF_4, LF_5, LF_6, LF_7, LF_8, LF_9): [Accuracy: $F(6, 108) = 2.05$, $p = 0.07$, $\eta_p^2 = 0.06$; Speed: $F(6, 108) = 1.75$, $p = 0.12$, $\eta_p^2 = 0.08$].

Table S1. Means (SDs) of reading accuracy and speed for each version of the tasks (TEXT, HF, LF, and NW).

**Reading Tasks**		**#1**	**#2**	**#3**	**#4**	**#5**	**#6**	**#7**	**#8**	**#9**	**#10**
**TEXT**	Accuracy[a]	99.29 (0.85)	99.49 (0.54)	99.39 (0.79)	99.51 (0.48)	99.42 (0.49)	99.48 (0.68)	99.33 (0.74)	99.48 (0.54)	99.29 (0.79)	99.68 (0.45)
	Speed[b]	5.07 (1.14)	5.28 (1.31)	5.16 (1.10)	5.38 (1.02)	5.43 (1.03)	5.05 (1.03)	5.50 (1.01)	5.71 (1.09)	5.41 (1.03)	5.34 (1.23)
**HF**	Accuracy[c]	0 (0)	0.17 (0.74)	0 (0)	0.33 (1.49)	0.67 (1.74)	0.67 (1.74)	0.67 (1.38)	0.17 (0.74)	0.17 (0.74)	
	Speed[b]	4.75 (0.79)	4.79 (1.02)	5.23 (1.28)	4.84 (0.84)	4.71 (1.02)	5.01 (1.17)	4.63 (0.93)	4.88 (0.98)	5.30 (0.94)	
**LF**	Accuracy[c]	0.52 (1.26)	0.17 (0.77)	0.34 (1.06)	0.52 (1.26)	1.21 (2.31)	0.69 (1.80)	1.21 (2.02)	1.55 (2.09)	1.55 (3.06)	
	Speed[b]	4.87 (0.98)	5.02 (0.96)	4.77 (1.19)	4.60 (1.21)	4.37 (1.13)	4.65 (1.05)	4.57 (0.93)	4.71 (1.22)	4.46 (1.15)	
**NW**	Accuracy[c]	5.00 (6.71)	3.33 (4.05)	2.83 (4.36)	3.50 (4.11)	4.17 (5.28)	4.00 (3.52)	3.33 (4.83)	3.83 (5.10)	3.50 (4.39)	
	Speed[b]	2.69 (0.70)	2.51 (0.69)	2.55 (0.86)	2.50 (0.73)	2.54 (0.77)	2.57 (0.71)	2.51 (0.68)	2.53 (0.66)	2.54 (0.71)	
**[a] Percentage (%) of accuracy, calculated as accuracy/total number of words x 100; [b] Syllables/seconds; [c] Percentage (%) of errors, calculated as errors/total number of words x 100. HF, High-Frequency words; LF, Low-Frequency words; NW, Non-words.**											

S.4 Safety, tolerability, and blinding assessment

Questionnaire of sensations related to transcranial electrical stimulation (TES)

(To be filled in by the participants and by the investigator)

Investigator:

Participant name/code: ___________________________________**Date:** ____/____/____

Experiment/Treatment: ______________________________

No stimulation experienced before [ ] **Experienced** [ ]

# of stimulation sessions before: ………………….

Type of electrical stimulation used here _______________**Intensity** __________mA (if known)

Electrodes dimensions: anode (if known)___*___ cathode (if known) ___*___ (shape_________)

other________________

Participant:

Did you experience any discomfort during the electrical stimulation? Please indicate the degree of intensity of your discomfort accordingly with the following scale:

- <u>**None**</u>: I did not feel the sensation addressed
- <u>**Mild**</u>: I mildly felt the sensation addressed
- <u>**Moderate**</u>: I felt the sensation addressed
- <u>**Strong**</u>: I felt the sensation addressed to a considerable degree

During stimulation session, I felt				
	None	*Mild*	*Moderate*	*Strong*
Headache	[ ]	[ ]	[ ]	[ ]
Neck pain	[ ]	[ ]	[ ]	[ ]
Scalp pain	[ ]	[ ]	[ ]	[ ]
Tingling	[ ]	[ ]	[ ]	[ ]
itching	[ ]	[ ]	[ ]	[ ]
Burning sensation	[ ]	[ ]	[ ]	[ ]
Skin redness	[ ]	[ ]	[ ]	[ ]
Drowsiness	[ ]	[ ]	[ ]	[ ]
Concentration difficulties	[ ]	[ ]	[ ]	[ ]
Severe mood changes	[ ]	[ ]	[ ]	[ ]
Other:_________	[ ]	[ ]	[ ]	[ ]

In case of perceived sensations, when did it begin?

[ ] At the beginning [ ] At approximately in the middle [ ] Towards the end of the stimulation

Duration (multiple options allowed)

[ ] Only initially [ ] It stopped in the middle of the block [ ] It stopped at the end of the block

How much did these sensations affect your general state?

[ ] Not at all [ ] Slightly [ ] Considerably [ ] Much [ ] Very much

Location of sensations:

[ ] Diffuse [ ] Localized [ ] Closed to the electrode, (which one?)____________ [ ] Other

If you would like to provide more details, please briefly describe the experimented sensations in relation to the “Other” or “Fatigue” or…. Response:

<u>To be administered at the end of each stimulation session:</u>

Do you believe that you received a real or a placebo stimulation?

[ ] Real [ ] Placebo [ ] I do not know

<u>Investigator:</u>

Please report any adverse event/problem that occurred and rate the event/problem on a scale from 0 to 3 as previously described.

____

____

____

____

Additional comments:

S4. References

1. Antal, A., Alekseichuk, I., Bikson, M., Brockmöller, J., Brunoni, AR., Chen, R., et al. (2017). Low intensity transcranial electric stimulation: Safety, ethical, legal regulatory and application guidelines. *Clinical Neurophysiology,* 128(9):1774–809.
2. Calvino, I. (1963). Marcovaldo. Torino: Einaudi.
3. Barca, L., Burani, C., & Arduino, L. S. (2002). Word naming times and psycholinguistic norms for Italian nouns. *Behavior Research Methods, Instruments & Computers,* 34(3), 424–434.
